# KEEPING COMMUNITY DURING A PANDEMIC: LGBTQ+ OLDER ADULTS AND THE VIRTUAL SENIOR CENTER

**DOI:** 10.1093/geroni/igac059.812

**Published:** 2022-12-20

**Authors:** Suzanne Marmo, Manoj Pardasani, David Vincent

**Affiliations:** Sacred Heart University, Fairfield, Connecticut, United States; Adelphi University, Garden City, New York, United States; SAGE Advocacy and Services for LGBTQ+ Elders, New York, New York, United States

## Abstract

During the initial stages of the pandemic, 96% of all senior centers ceased in-person programming, leaving many older adults without resources for meals, socialization, and critical services (NCOA, 2020). As a result of this shutdown, risk factors such as being a member of the underserved LGBTQ+ community, identifying as part of a racial or ethnic minoritized group, and/or experiencing poverty contributed to an increased likelihood of experiencing difficulties in meeting basic needs, reduced immunity to COVID and experiencing isolation (Berg-Weger and Morley, 2020; Kuehn, 2021). Despite the closure of many senior centers, some organizations were well positioned to strategically utilize pre-existing resources to help the community (Pendergrast, 2021). One organization, SAGE Advocacy and Services for LGBTQ+ Elders, the first publicly funded senior advocacy organization for LGBTQ+ older adults in the US, was one of the first to effectively transition to becoming a virtual senior center within days after the start of the pandemic (NYC DOA, 2020). Having a group of front-line workers who were highly embedded in their community, helped facilitate effective organizational adaptation and transition to a virtual senior center. This presentation seeks to describe how staff and program facilitators became vital resources for maintaining connection to the community of LGBTQ+ older adults. Focus groups with SAGE senior center employees and program facilitators were conducted in summer of 2021. Data identified resiliencies and barriers for maintaining community, providing vital services, and mitigating isolation with LGBTQ+ elders. Lessons learned and implications for organizations facing crises will be shared.

